# Peadiatric wheat challenge study-preliminary results

**DOI:** 10.1186/2045-7022-1-S1-P64

**Published:** 2011-08-12

**Authors:** Nora Nilsson, Gunilla Hedlin, Caroline Nilsson, Magnus Borres

**Affiliations:** 1 Astrid Lindgren Hospital, Karolinska Institute, Allergi, astma, Stockholm, Sweden; 2 Karolinska Institutet, Department of Woman and Child Health and Centre for Allergy Research, Stockholm, Sweden; 3 Karolinska Institutet and Sachs′ Children’s Hospital, Department of Clinical Science and Education Södesjukhuset, Stockholm, Sweden; 4 Sahlgrenska Academy of Göteborg University, Department of Pediatrics, Göteborg, Sweden

## Background

In addition to cow's milk, egg, soy, peanut, tree nuts and fish, wheat is also a food causing allergy in children. Wheat allergy is more prevalent in the northern part of Europe. Methods commonly used today in clinical practice to diagnose wheat allergy have a limited value in predicting a clinically significant wheat allergy compared with the test results for egg, milk and peanuts.

## Aim

To investigate what proportion of children with a diagnose of wheat allergy exhibit clinical symptoms upon oral food challenge with wheat and to identify if some children unnecessary avoid wheat.

## Material and Methods

27 children from Stockholm sensitized to wheat and subject to an elimination diet were tested for IgE antibodies to: wheat and ω-5 gliadin. All 27 patients were positive to IgE antibodies to wheat and underwent food challenge test. The initial oral food challenge test consists of 0,05gr of bread which is followed by six steps with increasing dose. The final dose is 17 gr of bread. The time interval between each dose is 30 min.

## Results

All 27 children presented different levels of IgE to wheat. 13 of them reacted during oral food challenge with clinical symptoms: 7 asthma attack, 10 urtikaria, 7 abdominal symptoms. We observed higher IgE levels to wheat in children who presented clinical symptoms during food challenge and additionally most of them showed to be positive to IgE antibodies to ω-5 gliadin. We also observed that higher levels of IgE to wheat were correlated to reactions to lower doses of wheat.

Our findings suggest that IgE antibodies to wheat alone cannot predict the outcome of food challenge and additional markers, such as ω-5 gliadin should be identified in order to improve the diagnostic workup for wheat allergy.

